# Dike intrusions during rifting episodes obey scaling relationships similar to earthquakes

**DOI:** 10.1038/srep03886

**Published:** 2014-01-28

**Authors:** Passarelli L., Rivalta E., Shuler A.

**Affiliations:** 1 Section 2.1 “Physics of earthquakes and volcanoes” GFZ-German Research Centre for Geosciences in plural form, Helmholtzstr. 7, 14467, Potsdam, Germany; 2 Department of Earth and Planetary Sciences, University of California Santa Cruz, 1156 High Street, Santa Cruz, CA 95064, USA; 3 Now at Chevron Energy Technology Company, 1500 Louisiana St., Houston, TX 77002, USA

## Abstract

As continental rifts evolve towards mid-ocean ridges, strain is accommodated by repeated episodes of faulting and magmatism. Discrete rifting episodes have been observed along two subaerial divergent plate boundaries, the Krafla segment of the Northern Volcanic Rift Zone in Iceland and the Manda-Hararo segment of the Red Sea Rift in Ethiopia. In both cases, the initial and largest dike intrusion was followed by a series of smaller intrusions. By performing a statistical analysis of these rifting episodes, we demonstrate that dike intrusions obey scaling relationships similar to earthquakes. We find that the dimensions of dike intrusions obey a power law analogous to the Gutenberg-Richter relation, and the long-term release of geodetic moment is governed by a relationship consistent with the Omori law. Due to the effects of magma supply, the timing of secondary dike intrusions differs from that of the aftershocks. This work provides evidence of self-similarity in the rifting process.

There is a general consensus that earthquakes exhibit self-similar behavior over a wide range of magnitudes on both local and global scales. Tectonic earthquakes satisfy two power-law scaling relationships to a reasonable approximation: the Gutenberg-Richter relation^1^,^2^, which describes the frequency-magnitude distribution, and the modified Omori law^3^,^4^, which describes the temporal decay of aftershocks. Driven by the accumulation of tectonic stresses, the earthquake rupture process is controlled by local stress conditions and the frictional and material properties of faults.

Self-similarity is also observed in volcanic systems. Both the global rate of volcanic activity^5^ and the frequency-volume distributions of major volcanic eruptions^6^ have been shown to follow power laws. Most volcanic eruptions are initiated by dike intrusions, which nucleate when the pressure inside an inflating magma reservoir exceeds a threshold value. Once a dike is nucleated, propagation is controlled by the reservoir pressure as well as viscous, elastic and thermal stresses^7^. Magma replenishment is necessary for additional intrusions to be sourced from the same reservoir^8^.

Along late-stage continental rifts, plate boundary separation is accommodated primarily by dike intrusions, and to a lesser extent by faults, during discrete rifting episodes^9^. Tensile stresses accumulated over decades to centuries exert the dominant controls on the deformation cycle, although crustal magma accumulation influences the timing of individual dike intrusions^9^,^10^,^11^,^12^,^13^. Discrete rifting episodes have been observed along two subaerial divergent plate boundaries^9^ - the Krafla segment of the Northern Volcanic Rift Zone in Iceland (1975–1984) and the Manda-Hararo segment of the Red Sea Rift in Ethiopia (2005–2010). In both episodes, the spatial and temporal patterns of dike intrusions share many similarities with tectonic earthquakes in mainshock-aftershock sequences. The initial and largest dike intrusions were followed by clusters of smaller dike intrusions. Additionally, in a process that closely resembles the triggering of aftershocks by static stress changes, later dike intrusions were emplaced in areas where local tectonic stresses had been either increased or not completely relieved by earlier dike intrusions^13^,^14^.

In the East African Rift System, the spatial distributions of earthquakes and volcanic vents are known to follow power laws^15^,^16^. Here, we examine the scaling relationships for dike intrusions associated with the Krafla and Manda-Hararo discrete rifting episodes. We perform a detailed statistical analysis of the dimensions and timings of individual dike intrusions and assess whether their behavior follows power laws analogous to the Gutenberg-Richter relation and the modified Omori law.

## Data

**Figure 1:**
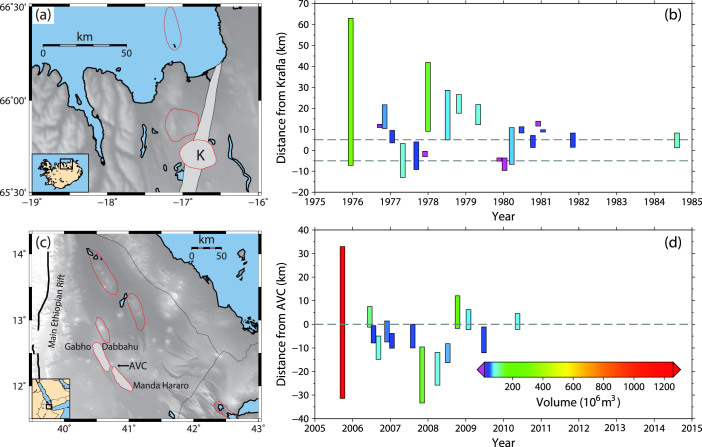
Summary of the Krafla and Manda-Hararo rifting episodes. (a), Map of the Northern Volcanic Rift Zone in Iceland. Thin red lines outline volcanic complexes and the Krafla caldera (K). (b), Location of dike intrusions as a function of time for the Krafla rifting episode. Colors indicate dike volumes in 10^6^ m^3^ and the dashed lines indicate the boundaries of the Krafla caldera. (c), Map of the Afar depression with political boundaries drawn as thin black lines. Thin red lines outline rift segments, with the Manda-Hararo rift segment shaded in light grey. Also shown are the Gabho and Dabbahu volcanoes and the Ado'Ale Volanic Complex (AVC). (d), Location of dike intrusions as a function of time for the Manda-Hararo rifting episode (MHA dataset). The dashed line denotes the location of the AVC axial magma chamber and the color scheme is the same as in (b). Panels (a) and (c) are created using the Generic Mapping Tools^55^ and panels (b) and (d) are modified after Wright *et al*^9^.

Our analysis is based on published records of dike intrusions associated with the Krafla and Manda-Hararo rifting episodes (Fig. 1 and Supplementary Information).

The Krafla rifting episode is composed of 20 major dike intrusions associated with subsidence of the Krafla caldera. Accounting for the fact that reservoirs expand as their internal pressures decrease, the volume of each dike intrusion is estimated to be twice the subsidence volume measured from elevation and gravity data (Fig. 1d)^17^,^18^. The lengths of the Krafla dike intrusions are constrained by field measurements of fractures and the hypocenters of triggered earthquakes^17^,^18^.

The dimensions of the 14 dike intrusions of the Manda-Hararo rifting episode are constrained well by seismic and geodetic observations^13^,^14^,^19^. We use the MHA^19^ (and I. Hamling personal communication 2011), and MHB^13^ datasets for estimates of the lengths and volumes of each dike intrusion. The MHB dataset does not include information for the most recent intrusion, but does provide estimates of the dislocated areas of the first thirteen dike intrusions. For the volume of the first intrusion in the MHB dataset, we exclude contributions from magma chambers beneath Dabbahu and Gabho volcanoes, and only consider the volume sourced from the magma reservoir associated with the Ado'Ale Volcanic Complex (Fig. 1a), which was active during the entire rifting episode^9^,^20^.

In addition to examining the dimensions of the dikes associated with each rifting episode separately, we also combine the volume information to produce two global rifting datasets - KMHA (Krafla and MHA) and KMHB (Krafla and MHB).

## A Gutenberg-Richter relation for rifting episodes

Empirically, tectonic earthquakes follow the Gutenberg-Richter relation^1^, *N*(*M*) = 10*^a^*^ − *bM*^, where *N(M)* is the number of earthquakes with magnitudes greater than or equal to *M* occurring in a given time, and *a* and *b* are constants describing earthquake productivity and the relative distributions of small and large earthquakes, respectively. Rewritten in terms of seismic moment, *M_0_*, the Gutenberg-Richter relation can be transformed into a probability density function^21^ 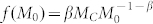 where *M_C_* is the seismic moment corresponding to the magnitude of completeness for a given seismicity catalog and *β* = *2/3* for the typical situation where the *b*-value is one^22^,^23^. The probability that an earthquake meets or exceeds a given size is therefore described by the survivor function *F*(*M*_0_) = (*M_C_*/*M*_0_)*^β^*. Because seismic moment is proportional to rupture area, if earthquakes can be approximated by slip on circular fault patches, similar power-law relationships can be defined for rupture area and length^24^.

The size of a dike intrusion is often described by the geodetic moment, which is proportional to the volume of magma intruded in an opening tensile crack. If dike intrusions along divergent plate boundaries obey self-similar scaling relationships like earthquakes, and if dike intrusions can be approximated by circular cracks, then the lengths, dislocated areas and volumes of dike intrusions should all follow power law distributions.

Here, we examine each of our rifting datasets for power-law behavior. Assuming that dike intrusions obey self-similar scaling relationships above some threshold size, *x_min_*, we model each survivor function as *N*(*X* ≥ *x*) = (*x*_min_/*x*)*^β^*, where *N*(*X* ≥ *x*) is the probability that a dike intrusion meets or exceeds a given size and *x* represents either the volumes, dislocated areas, or lengths of dike intrusions in a given rifting dataset. We estimate the parameters *β* and *x_min_* following Clauset *et al*^25^ and assess the goodness-of-fit using the Kolmogorov-Smirnov statistic. Using the value of *x_min_* determined from the power-law parameterization, we also model each rifting dataset with exponential and log-normal distributions and use the likelihood ratio test to determine which right-skewed distribution provides the best fit to the data (see Method and Tab. 1).

**Figure 2:**
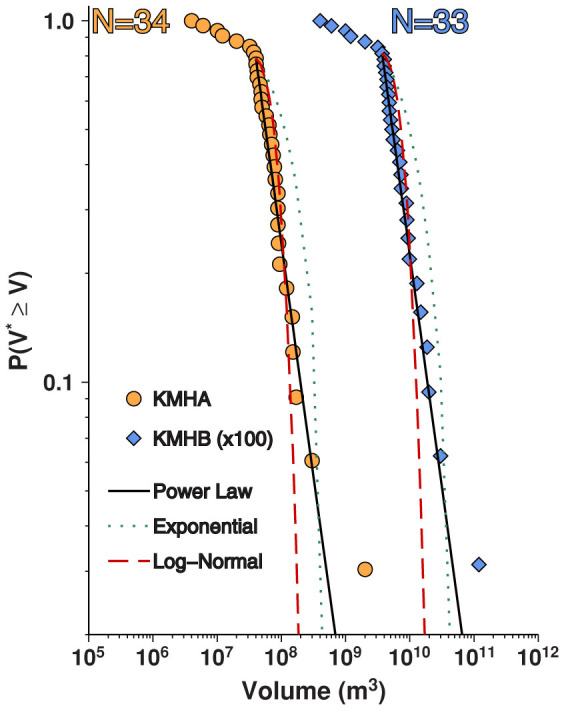
Survivor functions for the global rifting datasets. The KMHA dataset is drawn as orange circles and the KHMB dataset, multiplied by a factor of 100, is drawn as blue diamonds. The best-fit power laws are drawn as solid black lines. Best-fit log-normal and exponential distributions are drawn as dashed red lines and dotted green, respectively. The number of dike intrusions in each dataset is also reported.

**Figure 3:**
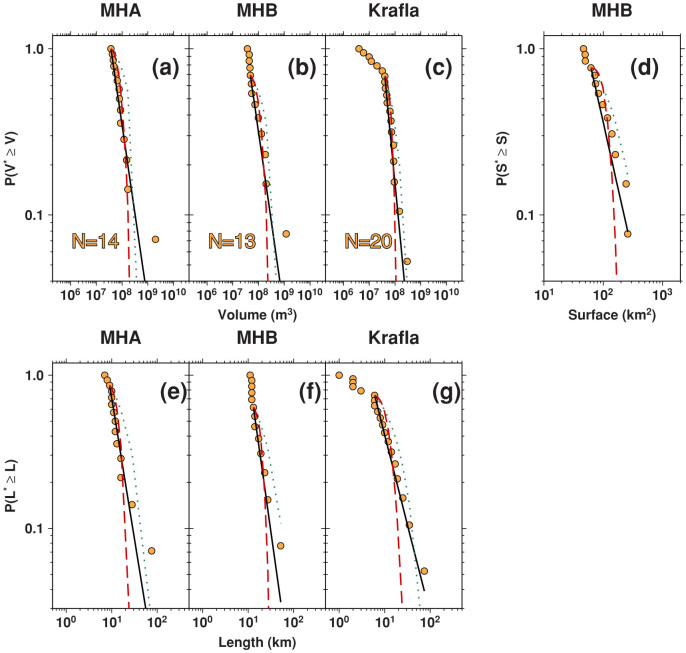
Survivor functions for individual rifting datasets. As in Fig. 2, survivor functions are plotted as orange circles, with solid black lines indicating to the best-fit power law and dotted green and dashed red lines indicating the best-fit exponential and log-normal parameterizations. Panels (a), (b), and (c) are for the MHA, MHB and Krafla volume datasets, panel (d) is for the MHB dislocated area dataset, and panels (e), (f), and (g) are for the MHA, MHB and Krafla length datasets, respectively. The number of events in each rifting dataset is also reported.

For the two global rifting datasets (KMHA and KMHB), we find that intrusion volumes are modeled well by power laws and poorly by exponential and log-normal distributions (Fig. 2). In most cases, power laws also outperform the alternative distributions for individual volume, length and area datasets (Tab. 1 and Fig. 3), although the small sample sizes preclude a statistically robust comparison. Nonetheless, our observation that a power law consistently performs well across the various rifting datasets suggests that dike intrusions have self-similar scaling relationships.

Power-law parameterizations for the global rifting datasets are characterized by *β*-values of ~1.3 to ~1.5, which are significantly higher than the theoretical value of *β* = *2/3* for earthquakes as well as the empirical value of *β* found for most tectonic earthquake sequences^2^,^26^,^27^. Earthquake sequences with elevated *β*-values (i.e. 1 < *β* < *2*), which have a larger proportion of small events, have been observed along divergent plate boundaries as well as in volcanic and geothermal environments^23^,^28^,^29^, and have been attributed to low values of confining stress^30^, material heterogeneity^31^, large thermal gradients^32^, and the presence of fluids^33^.

We note that the value of *x_min_* for both global rifting datasets is ~40 ×10^6^ m^3^ to ~60 ×10^6^ m^3^. Due to differences in how dike intrusions were detected at Krafla and Manda-Hararo, however, it is difficult to determine if *x_min_* results from catalog incompleteness^34^ or whether this quantity is associated with some threshold property of the source magma chambers, such as a minimum volume or pressure required for dike nucleation^7^,^35^.

Individual dike intrusions of the Krafla rifting episode were identified by rapid subsidence of Krafla caldera caused by the deflation of a 3–5 km deep magma chamber^17^. There, we note that the volume rifting datasets (Fig. 3c) could be fit by two power laws with different values of *β* corresponding to intrusions smaller and larger than *x_min_*. The smallest intrusions occurred later in the sequence within 10 km of the center of the caldera (Fig. 1d), and were accompanied by fissure eruptions. Our observation of a potential piecewise power-law relationship may be due to the fact that lateral propagation played a lesser role in the later dike intrusions, or because these events were controlled to a greater degree by local stresses rather than tectonic extension^9^.

At Manda-Hararo, individual dike intrusions were identified by local seismic and satellite geodetic data rather than monitoring the 8–10 km deep source magma chamber directly^9^, and we find that the volume, length and surface area rifting datasets are well modeled by a single power-law relationship. The values of *x_min_* for these datasets are close to the smallest dimensions observed, although we cannot exclude the possibility that additional small dike intrusions are missing from the catalog since the resolution of geodetic data decreases with depth and the maximum magnitude of earthquakes induced by dike intrusions is correlated with the volume of magma injected in the rift^36^.

## A modified Omori law for rifting episodes

For earthquakes, the decay of aftershock activity with time follows an empirical relationship known as the modified Omori law^3^, 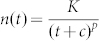, where *n*(*t*) is the rate of aftershocks in a given magnitude range occurring at time *t* since the mainshock, and *K*, *c*, and *p* are constants. Assuming that aftershocks follow the Gutenberg-Richter relation and the modified Omori law, the cumulative seismic moment released in a time interval can be expressed as the product of the average seismic moment released per event and the total number of events in the time interval, the integral form of the modified Omori law. It follows therefore that the seismic moment release rate also exhibits power-law time dependence^37^,^38^. These simple relationships result from self-similarity in the processes associated with static and dynamic earthquake triggering^39^,^40^, and deviations are often linked to the occurrence of strong aftershocks that generate secondary sequences of dependent events^3^.

A modified Omori law has also been identified for earthquakes preceding and following volcanic eruptions, which suggests that the brittle crust may respond similarly to tectonic and volcanic stresses^41^. Here, we evaluate whether the rate of volume emplaced in the crust during the rifting sequences can be modeled with power laws similar to the modified Omori law for earthquakes.

**Figure 4:**
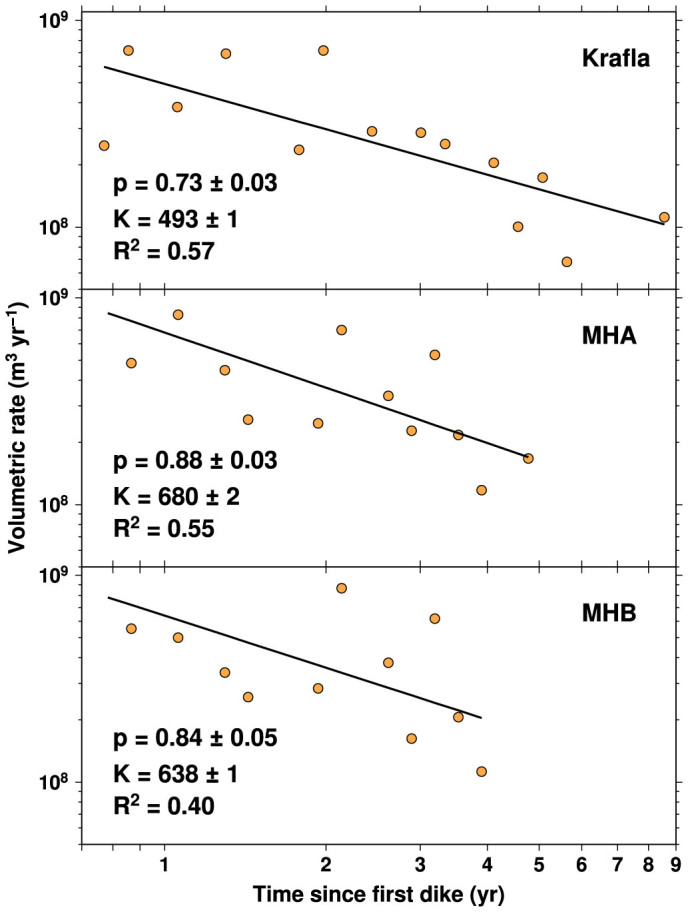
Omori-type decay of dike intrusion volume. Beginning after the initial dike intrusion in each rifting episode, the rate of volume emplacement as a function of time is indicated by orange circles. The upper panel is for the Krafla dataset, and the bottom two panels are for the MHA and MHB datasets, respectively. Black lines indicate *v_r_* = *K/t^p^* with the reported values of the best-fit parameters. The errors associated with *p* and *K* and the R^2^ value are also provided on each panel. The slopes of the regression lines are significantly different from zero at the 95% confidence level.

Analogous to earthquakes in a mainshock-aftershock sequence, the long-term volumetric rate during rifting episodes decays with time whereas the time between consecutive dike intrusions increases with time (Fig. 1). Secondary diking sequences are also observed following the largest dike intrusions. We quantify this behavior by calculating rifting volumetric rates using the equation *v_r_* = *K*/*t^p^*, where *t* is the time from the onset of a rifting episode, *p* is the power-law exponent and *K* is a constant (Fig. 4). From a simple linear regression in logarithmic space, we find that ~40–60% of the variance in the datasets can be explained by power-law relationships with *p*-values of 0.73 for Krafla and 0.84–0.89 for Manda-Hararo. These parameterizations closely resemble the form of the modified Omori law, which is predicted by rate-state theory for any impulsive stress change^42^. The relatively short emplacement times of the dike intrusions, which were on the order of a few hours to days, compared to interevent times of a few months to years may explain why we find consistency between the temporal behavior of earthquakes and dike intrusions in rifting episodes.

For earthquake sequences that follow the Gutenberg-Richter relation and the modified Omori law, it has been shown that aftershock productivity is a function of mainshock magnitude^43^. If rifting episodes are governed by similar physics, this could explain why the initial dike intrusions of the Krafla and Manda-Hararo sequences, which had volumes larger than ~100 ×10^6^ m^3^, were followed by several smaller diking events. Sequences with multiple dike intrusions and large cumulative volumes have also been recognized during past rifting episodes in the Northern Iceland Volcanic Zone^17^. Conversely, single dike intrusions are associated with rifting episodes at Dallol in Ethiopia in 2004 and Lake Natron in Tanzania in 2007. The volumes emplaced during the Dallol and Lake Natron episodes, 60 ×10^6^ m^3^ and 90 ×10^6^ m^3^ respectively^44^,^45^, are comparable to the values of *x_min_* for the Krafla and Manda-Hararo rifting datasets.

At volcanoes, the interevent repose time, or the time between magmatic eruptions, is correlated with erupted volume^46^. In open conduit systems characterized by short repose periods, volcanic eruptions generally follow the time-dependent model, where interevent time is dependent on both the size of the last eruption and the magma recharge rate^47^,^48^,^49^. Here, we investigate the role of the magma recharge rate in controlling the timing of individual dike intrusions at Krafla and Manda-Hararo by examining the time intervals between successive dike intrusions in each rifting episode. We use the Bayesian hierarchical time-predictable model of Passarelli *et al*^49^, assuming that the interevent time, *r_i_* = *t_i + 1_* − *t_i_*, follows the empirical equation *r_i_* = *δ v_i_^γ^*, where *v_i_* is the volume of the i*th* dike intrusion, and *δ* and γ are constants. If *γ* = 0 the system is not time-predictable, whereas if *γ* = 1 the magma recharge rate is constant and the interevent times and volumes of dike intrusions scale linearly^50^. We perform 10,000 simulations of the statistical model to determine the value of *γ* for each rifting dataset.

**Figure 5:**
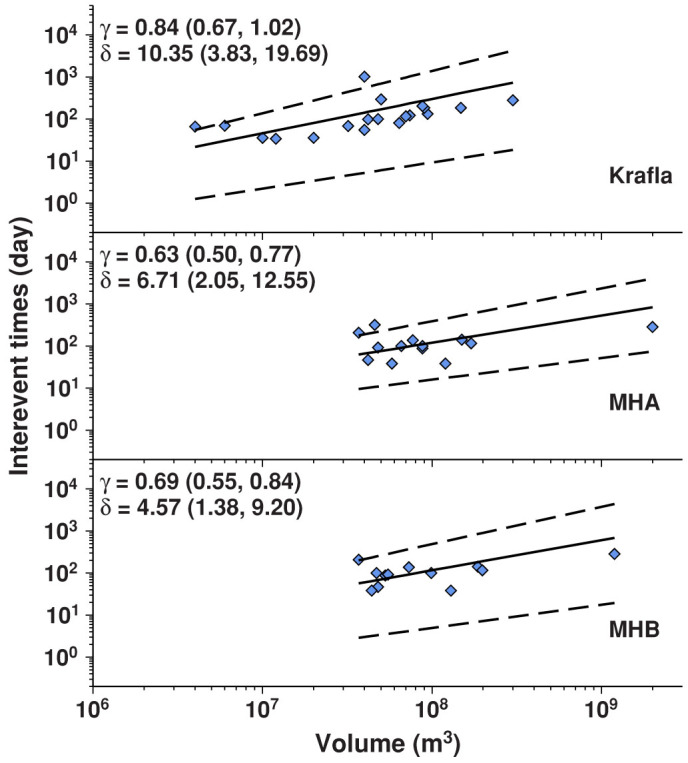
Time-predictable model for volume rifting datasets. For each volume dataset, blue diamonds indicate the time between successive dike intrusions plotted as a function of dike intrusion volume. Solid lines indicate the equation *r* = *δ v^γ^* with the best-fit curve obtained using the mean values of parameters *γ* and *δ*, which are reported in the top right of each panel. Dashed lines indicate the 5% and 95% confidence intervals for the best-fit curves, whose corresponding *γ and δ* values are reported in parentheses. The upper panel is the Krafla dataset and the bottom two panels are the MHA and MHB datasets. The units of *δ* are days per 10^6^ m^3^.

We find that the average values of γ are 0.84 for the Krafla dataset and 0.63 and 0.69 for the Manda-Hararo datasets (Fig. 5). These values of *γ* < 1 demonstrate a non-linear relationship between dike intrusion volume and interevent time, such that the interevent times following large dike intrusions are longer than those following small dike intrusions, but shorter than those predicted by the classical time-predictable model. Because the inflation of magma reservoirs is observed to decay exponentially after dike intrusions^8^,^20^,^51^, this observation suggests that the shallow magma chambers beneath Krafla and Manda-Hararo are hydraulically connected to larger and deeper reservoirs^9^ that allow magma recharge to occur at faster rates for larger pressure drops. The residuals about the best-fit regression lines for the Krafla and Manda-Hararo datasets are larger than those obtained for similar analysis completed for Kilauea and Etna volcanoes^49^, which may suggest that magmatic stresses play a lesser role in controlling the timing of individual dike intrusions during rifting episodes relative to purely volcanic systems.

Influenced by the magma recharge rate, the time-predictable behavior of dike intrusions at Krafla and Manda-Hararo seems to be in direct contrast to earthquakes, where the time interval between two events is inversely correlated with the magnitude of the first event, such that shorter interevent times are observed following larger earthquakes^52^,^53^.

## Discussion

We have demonstrated that the Krafla and Manda-Hararo diking episodes follow self-similar scaling relationships analogous to the Gutenberg-Richter relation and the modified Omori law for tectonic earthquakes. High *p_KS_*-values demonstrate the goodness of fit of the best-fit power-laws at high significance levels, and low *p_LR_*-values prove that power-laws reliably outperform the best-fit log-normal and exponential distributions even for our small sample sizes. Our statistical analysis demonstrates that the dimensions of dike intrusions follow a power-law model with small levels of variability around the best-fit parameters. With the possible existence of a pressure or volume threshold for dike nucleation, the major processes influencing the dimensions and timings of individual dike intrusions appear to be the accumulation of tectonic stress caused by long-term plate motion, static stress transfer between intrusions, and magma recharge.

Along continental rifts as well as mid-ocean ridges, dike emplacement is controlled by tectonic stresses and the magma supply rate. In areas with limited magma supply, where the pressure inside a crustal magma chamber is reduced during dike propagation, a sequence of dike intrusions rather than a single diking event may be necessary to fully release the accumulated tectonic stress^12^.

If the first dike intrusion encounters a structural barrier, it may continue to widen rather than lengthen until the driving pressure reaches a critical level, resulting in an aspect ratio that differs from later, smaller dikes intruded at lower magma pressures. Analogously, the thickness of the seismogenic zone limits the downdip rupture width, resulting in different aspect ratios for small and large earthquakes. The largest earthquakes are known to deviate substantially from the typical Gutenberg-Richter relation^54^, which is similar to our observation that the largest dike intrusions from the Krafla and Manda-Hararo rifting episodes do not obey the scaling laws that fit smaller dike intrusions well. We suggest that this discrepancy may be the result of factors such as the limited magma supply rate, threshold pressure conditions for dike propagation that change over time, and the structural constraints of lithospheric thickness and rift segment length.

Given that we are currently limited to studying rifting episodes along subaerial divergent plate boundaries, and since rifting episodes at a given location have recurrence intervals on the order of hundreds of years, we are restricted to this relatively small dataset. Nonetheless, the power-law behavior identified here is surprisingly robust, and with time and improvements in geophysical instrumentation, it may be possible to evaluate whether these observations apply more broadly to divergent plate boundaries in general.

## Methods

In order to estimate the parameters *β* and *x_min_* we follow the methodology presented by Clauset *et al*^25^ and select values that jointly minimize the Kolmogorov-Smirnov (KS) statistics. Errors for *β* and *x_min_* are estimated from 1000 bootstrap iterations^25^. Given the best-fitting values of *β* and *x_min_*, we calculate 1000 synthetic power-law distributed datasets and their KS statistics. The *p_KS_*-value associated with each rifting dataset is obtained by counting the fraction of the time that the KS statistics for the synthetic datasets are larger than the KS statistic for the empirical dataset. We rule out a power law distribution for datasets with *p_KS_*-value ≤ 0.1^25^.

We also model each rifting dataset using exponential and log-normal models left-truncated at *x_min_*, with the functional forms *f*(*x*) = *λ*exp(*λx_min_*)exp(−*λx*) and 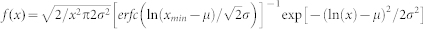, respectively. Once the best-fit distributions are found and the fits are assessed by the KS test as for the power law model, we use the likelihood ratio test for model selection. The logarithm of the likelihood ratio, *LR*, has a positive sign if the power-law distribution provides a better fit to the data than the alternative distribution. The associated *p_LR_*-value describes the statistical significance of *LR*, that is whether it is robustly far from zero. For large *p_LR_*-values, the likelihood ratio test cannot discriminate between the two distributions, however, for *p_LR_*-value ≤ 0.1, the sign of *LR* is a reliable indicator of which model provides a better fit to the data^23^.

Analogous to the classical Omori-decay fitting procedure, we bin the time intervals of each dataset (40 bins for MHA and MHB and 45 bins for Krafla) in order to obtain the rate of volume emplacement in each time interval. Bins with zero events are discarded. We use classical linear regression analysis to solve the equation *v_r_* = *K/t^p^* for the best-fit parameters and their associated errors, which are reported in Fig. 4 along with the *R^2^* value. We perform an F-test on the slope of the regression line where the null hypothesis is that the slope *p* = 0. The *p*-values are less than 0.02 in all three cases, which means we can reject the null hypothesis at the 95% confidence level.

We evaluate a time-predictable model for dike intrusions using the Bayesian hierarchical model developed in Passarelli *et al*^47^. We assume that the errors for the interevent times and volumes are one day and 25%, respectively. The posterior distributions of the γ and δ parameters are obtained by performing 10,000 simulations using the Metropolis-Hastings integration technique. Using these posterior distributions, we select the average values of the parameters as the best-fit values of the model and plot the 5*th* and 95*th* percentiles to demonstrate model variability.

## Author Contributions

L.P. and E.R. were involved in all stages of this study. A.S. contributed to the interpretation of the results and writing.

## Supplementary Material

Supplementary Information Supplementary information

## Figures and Tables

**Table 1:** Parameterizations of rifting datasets. For each of the nine rifting datasets, we report maximum likelihood estimates of power-law, exponential and log-normal parameterizations. p_KS_ values greater than 0.10 are reported in bold and indicate statistically significant results from the Kolmogorov-Smirnov test. Errors associated with β are one bootstrap standard deviation, and x_min_ values should be multiplied by 1 ×10^6^ m^3^ for volume datasets, 1 km^2^ for the dislocated area dataset, and 1 km for the length datasets. The final two columns show the results of the likelihood ratio test, which was only performed when the alternative distribution fits the data well. p_LR_ values less than or equal to 0.10 are reported in bold and indicate statistically significant results of the likelihood ratio test. See Methods

Rifting Dataset	Power Law			Exponential		Log-Normal			Power Law vs Exponential		Power Law vs Log-Normal	
	*β*	*x*_min_	p_KS_-value	*λ*	*p*_KS_-value	*μ*	*σ*	*p*_KS_-value	LR	*p*_LR_-value	LR	*p*_LR_-value
KMHA volumes	1.46 ± 0.49	58 ± 16	**0.35**	0.048	0.00	4.73	0.77	0.06	–	–	–	–
KMHB volumes	1.28 ± 0.64	37 ± 20	**0.83**	0.008	0.00	4.39	0.76	**0.15**	–	–	5.36	**0.09**
MHA volumes	1.06 ± 0.53	37 ± 11	**0.84**	0.048	0.08	4.54	0.96	**0.21**	–	–	3.65	**0.03**
MHB volumes	1.08 ± 0.57	48 ± 19	**0.91**	0.004	**0.19**	4.80	0.95	**0.33**	4.3	**0.03**	2.21	**0.10**
Krafla volumes	1.62 ± 0.52	40 ± 14	**0.53**	0.011	0.00	4.29	0.56	**0.43**	–	–	2.23	0.22
MHB areas	1.59 ± 0.94	62 ± 23	**0.52**	0.008	**0.34**	4.76	0.48	**0.68**	0.30	0.93	−0.63	0.77
MHA lengths	1.84 ± 0.50	9 ± 1	**0.57**	0.048	0.00	2.69	0.58	**0.12**	–	–	4.13	**0.00**
MHB lengths	2.11 ± 0.33	13 ± 2	**0.90**	0.045	0.00	3.00	0.43	**0.41**	–	–	1.74	0.11
Krafla lengths	1.17 ± 0.47	6 ± 3	**0.97**	0.056	0.00	2.56	0.72	**0.40**	–	–	2.55	0.13
