# Supplementary material for: Dike intrusions during rifting episodes obey scaling relationships similar to earthquakes
Source: Sci Rep. 2014 Jan 28;4:3886. doi: 10.1038/srep03886 (PMC3904149; doi:10.1038/srep03886)
Supplement: Supplementary Information [file srep03886-s1.pdf]

Dike intrusions during rifting episodes obey scaling relationships similar to earthquakes

Supplementary Information

Passarelli, L. [1], Rivalta, E., [1] & Shuler, A. [2,3]

[1]*Section 2.1 ”Physics of earthquakes and volcanoes” GFZ-German Research Centre for Geosciences, Helmholtzstr. 7, 14467, Potsdam, Germany*

[2]*Department of Earth Planetary Sciences, University of California Santa Cruz, 1156 High Street, Santa Cruz, CA 95064, USA*

[3]*Now at Chevron Energy Technology Company, 1500 Louisiana St., Houston, TX 77002, USA*

Data

The volume and length datasets for the 1975-1984 Krafla rifting episode are based on published records of individual ground deformation events. In Supplementary Table 1, we report volumes of subsidence of the Krafla caldera from Tryggvason (1984)[1] and Arnadottir *et al.* (1998)[2]. To obtain the volumes of dike intrusions associated with these deflation events, we multiply the subsidence volumes by two. The length dataset is based on digitization of Figure 2 in Buck *et al.* (2006)[3]. We note that this figure includes one dike intrusion that is not discussed in the detailed chronology of the rifting episode [3]. Correcting for this discrepancy (P. Einarsson personal communication, 2012), we exclude the extra intrusion and use the onset times of dike intrusions reported by Tryggvason (1984) [1].

Estimates of the dimensions of dike intrusions associated with the 2005-2010 Manda-Hararo rifting episode are available from multiple sources. In Supplementary Table 2, we report the MHA dataset, which contains estimates of volume and length, and the MHB dataset, which contains estimates of volume, length and dislocated area. Volumes for the MHA dataset are from Ebinger *et al.* (2010)[4] and I. Hamling (personal communication, 2011), and the lengths are digitized from Figure 2a in Wright *et al.* (2012)[5]. The MHB dataset is based on Table 1 and Figure 3 of Grandin *et al.* (2010)[6]. For the first dike intrusion, we use the sum of values given for the d0a and d0b dike segments since the northernmost part of the dike intrusion was likely sourced from magma reservoirs that were not active during the remainder of the rifting episode [6].

1. Tryggvason, E. Widening of the Krafla fissure swarm during the 1975–1981 volcano-tectonic episode. *Bulletin Volcanologique* **47**, 47–69 (1984).

2. Arnadottir, T., Sigmundsson, F. & Delaney, P. T. Sources of crustal deformation associated with the Krafla, Iceland, eruption of September 1984. *Geophys. Res. Lett* **25**, 1043–1046 (1998).

3. Buck, W., Einarsson, P. & Brandsdóttir, B. Tectonics stress and magma chamber size as controls on dike propagation: constraints from the 1975-1984 Krafla rifting episode. *J. Geophys. Res.* **111**, B12404 (2006).

4. Ebinger, C. *et al.* Length and timescales of rift faulting and magma intrusion: the Afar rifting cycle from 2005 to present. *Annu. Rev. Earth Planet. Sci.* **38**, 439–466 (2010).

5. Wright, T.J. *et al.* Geophysical constraints on the dynamics of spreading centres from rifting episodes on land. *Nature Geosci.* **5**, 242–250 (2012).

6. Grandin, R. *et al.* Sequence of rifting in Afar, Manda-Hararo rift, Ethiopia, 2005–2009: Time-space evolution and interactions between dikes from interferometric synthetic aperture radar and static stress change modeling. *J. Geophys. Res.* **115**, B10413 (2010).

Supplementary Table 1. Krafla data

[1]multiplied by two in the analysis

Krafla			
Date	Time since first dike (days)	Volume [1] ($10^6 m^3$)	Length (km)
20-Dec-1975	0	150	74
25-Sep-1976	280	10	1
31-Oct-1976	316	32	12
20-Jan-1977	397	21	6
28-Apr-1977	495	47	17
08-Sep-1977	628	20	14
02-Nov-1977	683	2	35
07-Jan-1978	749	74	25
10-Jul-1978	933	37	9
10-Nov-1978	1056	45	10
13-May-1979	1240	44	2
03-Dec-1979	1444	3	6
11-Feb-1980	1514	6	19
16-Mar-1980	1548	35	3
10-Jul-1980	1664	24	6
18-Oct-1980	1764	16	2
25-Dec-1980	1832	5	2
30-Jan-1981	1868	25	7
18-Nov-1981	2160	20	8
04-Sep-1984	3181	50	7

Supplementary Table 2. Manda-Hararo datasets

		MHA		MHB		
Date	Time since first dike (days)	Volume ($10^6 m^3$)	Length (km)	Volume ($10^6 m^3$)	Area (km^2)	Length (km)
04-Sep-2005	0	2000	76	1189	259.8	52
17-Jun-2006	286	120	10	129	118.9	14
25-Jul-2006	324	42	9	48	62.2	11
10-Sep-2006	371	88	12	53	98.4	13
07-Dec-2006	459	58	11	44	49.9	12
14-Jan-2007	497	37	7	37	72.5	14
11-Aug-2007	706	48	12	55	49.7	12
11-Nov-2007	798	150	28	186	242.9	23
31-Mar-2008	939	88	16	99	161.1	27
09-Jul-2008	1039	66	10	47	74.2	19
17-Oct-2008	1139	170	16	198	140	17
11-Feb-2009	1256	77	10	73	83.5	12
29-Jun-2009	1394	46	13	44	46.6	12
15-May-2010	1714	80	8			
